# DynamicFU: Contribution-Aware Dynamic Federated Unlearning for Industrial IoT

**DOI:** 10.3390/s26123714

**Published:** 2026-06-11

**Authors:** Ziang Wu, Buzhen He, Zhiwei Si, Xiuheng Liao, Chunhua Su

**Affiliations:** 1 Graduate School of Computer Science and Engineering, University of Aizu, Aizu-Wakamatsu 965-8580, Japan; d8262105@u-aizu.ac.jp (Z.W.); d8272107@u-aizu.ac.jp (Z.S.); chsu@u-aizu.ac.jp (C.S.); 2 School of Computer Science and Artificial Intelligence, Lanzhou University of Technology, Lanzhou 730050, China; 231080292006@lut.edu.cn

**Keywords:** Industrial Internet of Things, federated learning, federated unlearning, privacy-preserving

## Abstract

The Industrial Internet of Things (IIoT) increasingly relies on federated learning (FL) to enable collaborative model training without directly sharing raw traffic data across industrial sites. However, in practical IIoT deployments, clients may later request the removal of their data contributions from a trained federated model due to regulatory requirements, such as the General Data Protection Regulation (GDPR), ownership transfer, or internal data-governance policies. Such practical requirements create a strong demand for federated unlearning in IIoT applications. Furthermore, IIoT deployments often exhibit highly imbalanced client data distributions, resulting in substantially different contributions of individual clients to the global model. Nevertheless, most existing federated unlearning methods adopt a uniform unlearning strategy and fail to account for such client-level contribution gaps. To address this issue, we propose DynamicFU, a contribution-aware dynamic federated unlearning framework for IIoT deployments. The proposed method evaluates the target client from parameter-level, data-level, and performance-level perspectives and adaptively determines the unlearning strength by dynamically adjusting the number of unlearning rounds. Experimental results on public IIoT datasets show that DynamicFU substantially improves unlearning efficiency, achieving up to 22.89× speedup over Full Retrain while maintaining comparable effectiveness.

## 1. Introduction

Industrial Internet of Things (IIoT) systems are becoming a fundamental component of modern industrial infrastructure by enabling large-scale sensing, monitoring, control, and intelligent decision-making across distributed industrial environments. To support collaborative intelligence while avoiding direct raw-data sharing among industrial sites, federated learning (FL) has emerged as a practical paradigm for IIoT applications [1]. By keeping data locally and only exchanging model updates, FL offers an appealing balance between data utility and privacy preservation [2]. In practical IIoT deployments, each federated client may correspond to an industrial plant, an edge gateway, a production line, or a domain-specific monitoring system in scenarios such as smart manufacturing, energy systems, and industrial network monitoring. These clients often collect traffic and operational records under different device configurations, production schedules, and security policies. Therefore, directly centralizing industrial data is usually undesirable, as such data may reveal sensitive information about system topology, equipment behavior, and operational routines.

While federated learning keeps industrial data local during training, it does not by itself support post-training removal of learned data influence. Privacy regulations such as the General Data Protection Regulation (GDPR), especially the right to erasure, often referred to as the “right to be forgotten”, create a practical need for federated unlearning (FU), which aims to remove the influence of specified data, classes, or clients from trained federated models without full retraining [3,4]. In industrial environments, such removal requests may also arise when an edge site leaves a collaborative federation, when equipment is decommissioned or transferred to another operator, or when internal data-retention policies require previously contributed records to be excluded from deployed models. In these cases, simply deleting the raw local data is insufficient because the trained federated model may still encode the statistical influence of the removed client. In this work, we focus on client-level FU, where the objective is to remove the influence of a target client from the trained federated model. This is particularly important for IIoT systems, where continuous operation, limited maintenance windows, and high computation and communication costs make full retraining impractical.

Despite its promise, federated unlearning in IIoT remains challenging. A key difficulty is that IIoT clients usually exhibit highly heterogeneous data distributions and unequal contributions to the global model [5,6]. Some clients may provide larger data volumes, more informative patterns, or stronger utility gains, while others contribute much less [7]. Such contribution differences are natural in industrial federations, since clients may operate under different production loads, device types, attack exposure levels, and monitoring policies. Nevertheless, most existing FU methods adopt a uniform unlearning strategy for all target clients, ignoring these client-level contribution differences [8,9]. Such a one-size-fits-all design may lead to either insufficient unlearning for high-contribution clients or unnecessary utility degradation for lower-contribution clients. This issue is especially critical in IIoT environments, where statistical heterogeneity, deployment diversity, and operational constraints are common.

To address the above issues, we propose DynamicFU, a contribution-aware dynamic federated unlearning framework for IIoT. The proposed method evaluates the target client from multiple perspectives, including parameter contribution, data contribution, and performance contribution, and then adaptively determines the unlearning strength through dynamic unlearning rounds. In this way, the unlearning process becomes better aligned with the actual contribution of each client to the trained federated ensemble, improving the balance between unlearning effectiveness and system efficiency.

The main contributions of this paper are summarized as follows.

- A contribution-aware federated unlearning framework for IIoT is established, where client-level unlearning strength is no longer fixed but adaptively adjusted according to the target client’s contribution to the trained federated ensemble.
- A multi-perspective client contribution evaluation mechanism is introduced by jointly considering parameter-level, data-level, and performance-level contributions, enabling a more principled estimation of how much unlearning should be applied to each target client.
- A dynamic unlearning strategy is designed to convert the estimated client contribution into adaptive unlearning rounds, which improves unlearning efficiency while preserving model utility as much as possible.
- Extensive experiments on public IIoT datasets demonstrate that DynamicFU achieves effectiveness comparable to Full Retrain while significantly reducing unlearning cost, showing the practical potential of dynamic federated unlearning for IIoT systems.

The rest of this paper is organized as follows. Section 2 reviews related work. Section 3 formalizes the sharded federated learning and client-level unlearning protocol. Section 4 presents the proposed DynamicFU framework. Section 5 introduces the experimental setup and evaluation metrics. Section 6 reports and analyzes the experimental results. Section 7 discusses the results, and Section 8 concludes this paper.

## 2. Related Work

The studies most relevant to this work can be grouped into three directions: FL in IIoT, FU, and heterogeneity-aware FL. Existing studies provide important foundations for privacy-preserving distributed learning and efficient model removal, but client-level federated unlearning for IIoT under unequal client contributions remains insufficiently explored.

### 2.1. Federated Learning in IIoT

IIoT systems are typically characterized by distributed data ownership, privacy-sensitive industrial information, and heterogeneous local environments, which make centralized learning difficult to deploy in practice [10,11]. FL provides a natural solution by enabling collaborative model training without directly sharing raw local data [12]. This paradigm is particularly attractive for IIoT scenarios, where industrial participants may be unwilling or unable to upload local data to a central server [1,13].

Nevertheless, IIoT-oriented FL faces significant challenges due to statistical and system heterogeneity across clients [14,15]. Prior studies have shown that heterogeneous local data and device conditions can substantially affect optimization stability, communication efficiency, and model quality in federated systems [5,16]. Personalized federated learning further indicates that treating all clients identically is often suboptimal when local distributions differ substantially [17]. These observations imply that heterogeneity should also be carefully considered in post-training model update and removal tasks in IIoT.

### 2.2. Federated Unlearning

FU has emerged as an important direction for removing the influence of previously used data or participating clients from trained federated models [18]. Existing studies have explored different unlearning granularities, including sample-level, class-level, and client-level settings, while client-level unlearning is especially important for practical federated systems in which an entire participant may request removal [19].

Representative client-level FU methods have mainly focused on improving efficiency relative to full retraining. FedEraser reconstructs an unlearned model from stored historical updates [20], while Rapid Retraining methods aim to accelerate exact client removal [21]. Recent studies have further explored asynchronous unlearning, exact unlearning, clustered one-shot aggregation, sequential informed restart strategies, and communication-efficient provable removal [22,23,24,25,26,27]. Although these methods have substantially advanced the efficiency and rigor of federated unlearning, most of them still treat the unlearning target in a relatively uniform manner and do not explicitly adapt unlearning strength according to the target client’s actual contribution to the global model.

### 2.3. Heterogeneity-Aware and Adaptive Methods in Federated Learning and Unlearning

Extensive FL research has shown that client heterogeneity is a key factor shaping distributed model behavior [5]. In heterogeneous environments, clients may differ not only in data volume, but also in data quality, feature distribution, task difficulty, and optimization impact [28,29]. Consequently, their influence on the final federated model can be highly unequal. This insight suggests that post-training removal should not always follow a fixed or uniform unlearning strategy.

Some recent studies have begun to introduce adaptive or personalized mechanisms into federated unlearning. For example, Lin et al. explored incentive-driven dynamic client selection to improve the flexibility of the unlearning process [30]. Pan et al. further investigated personalized federated unlearning with an optimal incentive mechanism, allowing data owners to adjust unlearning intensity according to their requirements while balancing unlearning demands and model utility preservation [31]. However, existing adaptive designs still pay limited attention to a fundamental issue in IIoT environments, namely, that unlearning strength should be associated with the actual contribution of the target client to the trained global model. Therefore, a gap remains between heterogeneous IIoT federated systems and current federated unlearning methods.

Overall, existing studies provide a strong foundation for privacy-preserving distributed learning and efficient unlearning, but they do not sufficiently address client-level unlearning under unequal client contributions in heterogeneous IIoT environments. This gap motivates the proposed DynamicFU framework, which explicitly connects client contribution estimation with adaptive unlearning strength.

## 3. Federated Learning and Unlearning Protocol

This section formalizes the shard-based FL architecture adopted in this work and defines the corresponding client-level FU problem in IIoT environments. Different from standard federated learning with a single global model, the implementation used in this paper organizes clients into multiple shards, trains one federated model for each shard, and performs inference through an ensemble of shard models. Accordingly, the unlearning target is not a single model but a set of shard models.

### 3.1. Sharded Federated Learning Architecture

Let $C=\{c_{1},c_{2},\ldots ,c_{N}\}$ denote the set of participating IIoT clients, where client $c_{i}$ owns a private local dataset $D_{i}$ with size $n_{i}$, and let $n=\sum_{i=1}^{N}n_{i}$ denote the total number of training samples.

Instead of training a single global model over all clients, the client set is first partitioned into *S* disjoint shards:

(1) $$C=\overset{S}{\underset{s=1}{⋃}}C_{s},\;C_{a}\cap C_{b}=Ø\;\mathrm{for}\;a\neq b,$$

where $C_{s}$ denotes the client subset assigned to shard *s*, and $n_{s}=\sum_{c_{i}\in C_{s}}n_{i}$ is the total data size of shard *s*.

For each shard *s*, a separate federated model is trained by applying FedAvg only to the clients within that shard. Let $w_{s}^{t}$ denote the shard model at communication round *t*. If $P_{s}^{t}\subseteq C_{s}$ is the client subset participating in round *t*, the shard-level aggregation is

(2) $$w_{s}^{t+1}=\sum_{c_{i}\in P_{s}^{t}}\frac{n_{i}}{\sum_{c_{j}\in P_{s}^{t}}n_{j}}\;w_{s,i}^{t+1},$$

where $w_{s,i}^{t+1}$ is the locally updated model of client $c_{i}$ in shard *s*.

Accordingly, the optimization objective of shard *s* is

(3) $$\min_{w_{s}}F_{s}\left(w_{s}\right)=\sum_{c_{i}\in C_{s}}\frac{n_{i}}{n_{s}}F_{i}\left(w_{s}\right),$$

where

(4) $$F_{i}\left(w\right)=\frac{1}{n_{i}}\sum_{\left(x,y\right)\in D_{i}}\ell \;\left(f(x;w),y\right)$$

is the local empirical risk of client $c_{i}$, where ℓ(·,·) denotes the classification loss applied to logits and labels.

After training all shards, the overall federated model is represented as a set of shard models, denoted by $W^{*}=\left\{w_{1}^{*},w_{2}^{*},\ldots ,w_{S}^{*}\right\}$.

For an IIoT sample *x*, inference is performed by aggregating the logits produced by all shard models:

(5) $$z\left(x\right)=\sum_{s=1}^{S}f\left(x;w_{s}^{*}\right),$$

where $f(x;w_{s}^{*})$ denotes the logit output of shard model $w_{s}^{*}$. The final prediction probability is p(y=1∣x)=σ(z(x)), where σ(·) is the sigmoid function.

Therefore, this sharded-ensemble structure determines how client-level unlearning is formulated and implemented.

### 3.2. Client-Level Unlearning Problem Under Sharded Training

After training has converged, suppose a target client $c_{u}\in C$ requests that its contribution be removed. Let $s_{u}$ denote the shard containing $c_{u}$, i.e., $c_{u}\in C_{s_{u}}$.

After removing the target client, the updated client set of shard *s* is defined as

(6) $$C_{s}^{\setminus u}=\left\{\begin{array}{cc} C_{s}, & s\neq s_{u},\\ C_{s_{u}}\setminus \left\{c_{u}\right\}, & s=s_{u}. \end{array}\right.$$

The corresponding shard-level retraining objective becomes

(7) $$\min_{w_{s}}F_{s}^{\setminus u}\left(w_{s}\right)=\sum_{c_{i}\in C_{s}^{\setminus u}}\frac{n_{i}}{n_{s}^{\setminus u}}F_{i}\left(w_{s}\right),$$

where $n_{s}^{\setminus u}=\sum_{c_{i}\in C_{s}^{\setminus u}}n_{i}$.

A comprehensive offline reference is to retrain the sharded federated system from scratch after removing $c_{u}$, yielding

(8) $$W^{\setminus u}=\left\{w_{1}^{\setminus u,*},w_{2}^{\setminus u,*},\ldots ,w_{S}^{\setminus u,*}\right\}.$$

The goal of client-level federated unlearning is then to produce an unlearned ensemble ${\tilde{W}}^{\setminus u}$ that approaches this retraining-based reference while avoiding the cost of full retraining.

Compared with single-model formulations, the unlearning target here has two properties. First, only the shard containing the target client is directly affected by client removal. Second, the prediction behavior of the whole system still depends on the ensemble of all shard models. Therefore, effective client-level unlearning in this work should satisfy three requirements:

1. It should remove the contribution of the target client from the affected shard and the final ensemble prediction;
2. It should preserve the utility of the retained ensemble on the target IIoT task;
3. It should achieve the above goals at substantially lower cost than retraining all shards from scratch.

## 4. DynamicFU Framework

This section presents DynamicFU, which estimates the target client’s contribution and adaptively determines the retraining intensity of the affected shard after client removal.

### 4.1. Framework Overview

**Figure 1:**
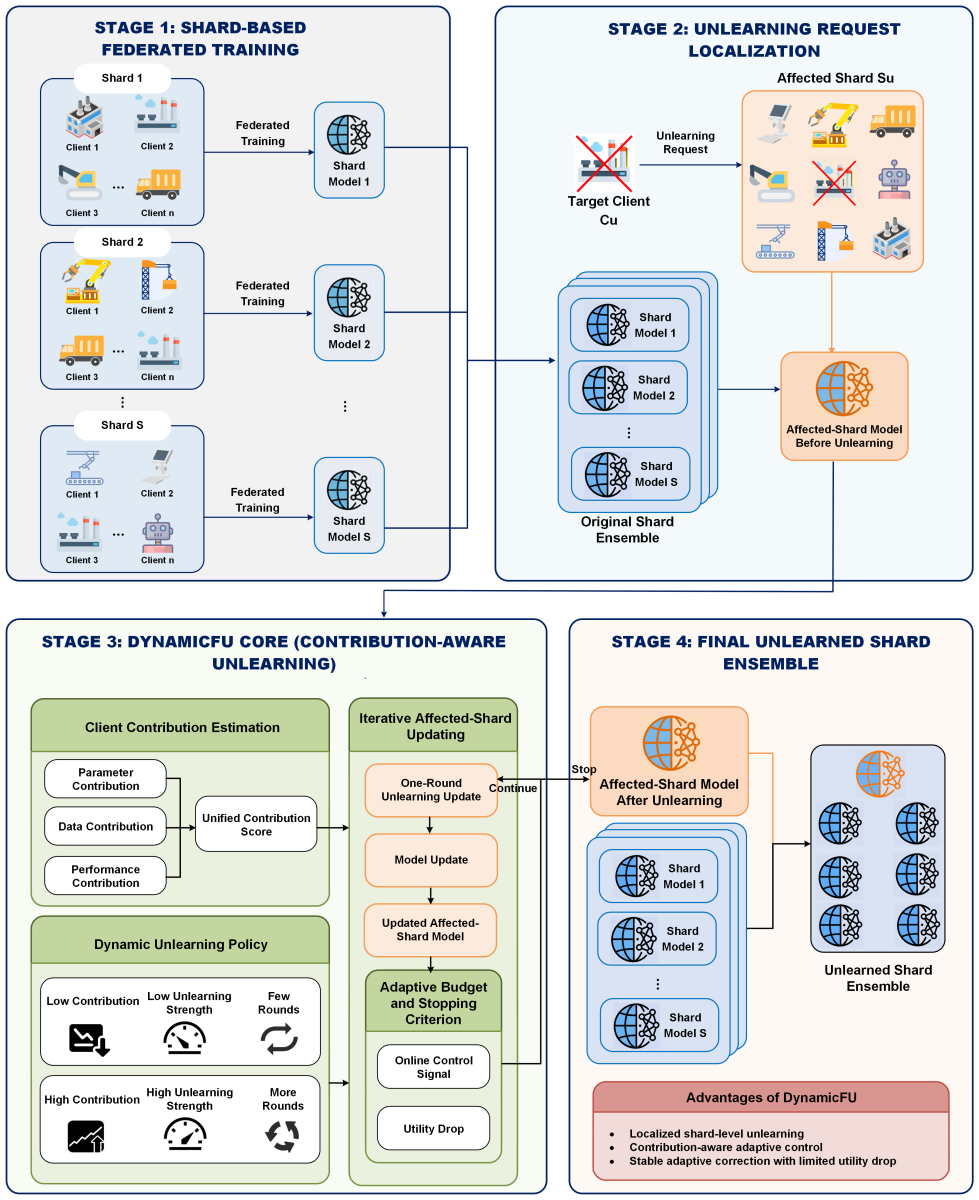
Overview of the proposed DynamicFU framework for client-level federated unlearning in IIoT environments.

As illustrated in Figure 1, DynamicFU starts from a trained shard-based federated ensemble $W^{*}=\left\{w_{1}^{*},\ldots ,w_{S}^{*}\right\}$. When a client requests removal due to privacy, regulatory, or operational reasons, the framework first identifies the shard containing the target client and then estimates the target client’s contribution from parameter-level, data-level, and performance-level perspectives. Based on the resulting unified contribution score, DynamicFU assigns an initial unlearning strength and adaptively retrains only the affected shard. This design avoids assigning the same unlearning intensity to clients with substantially different contributions.

### 4.2. Client Contribution Estimation

As the contribution-aware component of DynamicFU, this subsection characterizes the importance of the target client from three complementary perspectives: parameter contribution, data contribution, and performance contribution. Since these quantities have different scales, each component is first computed in raw form and then normalized across all candidate clients.

Different from uniform unlearning strategies that assign the same correction budget to every removal request, the proposed contribution formulation is designed to estimate the client-specific influence that should be removed after training. It is not a standalone data-valuation score or a static client-selection criterion. Instead, it jointly measures how much a client can perturb the trained shard model, how distinctive its local data distribution is, and how strongly its update changes validation behavior. The resulting score is then explicitly coupled with the unlearning strength and adaptive retraining budget. Therefore, the formulation directly connects client contribution estimation with post-removal unlearning intensity, which is the key distinction between DynamicFU and replay-based or fixed-budget federated unlearning baselines.

#### 4.2.1. Parameter Contribution

The parameter contribution reflects how strongly a client can perturb the current shard model. Let $c_{i}$ be a candidate client belonging to shard s(i), and let $w_{s(i)}^{*}$ be the trained model of that shard. Starting from $w_{s(i)}^{*}$, we perform a short local update using only the data of client $c_{i}$ and obtain an updated model $w_{s(i),i}^{+}$. The raw parameter contribution is defined as

(9) $$u_{i}={∥\mathrm{vec}\;\left(w_{s(i),i}^{+}\right)-\mathrm{vec}\;\left(w_{s(i)}^{*}\right)∥}_{2},$$

where vec(·) denotes vectorization of model parameters.

A larger $u_{i}$ indicates that the client can induce a stronger model shift and therefore has a higher contribution from the parameter-update perspective.

#### 4.2.2. Data Contribution

The data contribution is designed to capture both client data heterogeneity and task-relevant label characteristics. Let $p_{i}$ denote the binary label distribution of client $c_{i}$, and let $p_{\mathrm{train}}$ denote the label distribution of the overall training set. We measure distributional distinctiveness using the Jensen–Shannon (JS) divergence and further incorporate the attack-class ratio $\pi_{i}=n_{i}^{\mathrm{atk}}/n_{i}$. The raw data contribution is defined as

(10) $$d_{i}=\lambda_{\mathrm{JS}}\;\mathrm{JS}\left(p_{i}\;∥\;p_{\mathrm{train}}\right)+\lambda_{\pi }\pi_{i},$$

where $\lambda_{\mathrm{JS}},\lambda_{\pi }\geq 0$ and $\lambda_{\mathrm{JS}}+\lambda_{\pi }=1$.

This term reflects that a client may be influential because its label distribution or task-relevant label composition is more distinctive than that of other clients.

#### 4.2.3. Performance Contribution

The performance contribution quantifies how much the client can affect the utility of the ensemble. For client $c_{i}$, we construct a temporary ensemble by replacing the original shard model $w_{s(i)}^{*}$ with the one-step locally updated model $w_{s(i),i}^{+}$ while keeping all other shard models unchanged. Let the resulting ensemble be denoted by $W_{i}^{+}$. The raw performance contribution is defined as

(11) $$q_{i}=\left|F1_{\mathrm{macro}}\left(W_{i}^{+}\right)-F1_{\mathrm{macro}}\left(W^{*}\right)\right|,$$

where $F1_{\mathrm{macro}}\left(\cdot \right)$ is measured on the validation set.

A larger $q_{i}$ means that injecting the client-specific update changes the validation behavior of the ensemble more substantially, suggesting stronger task-level influence.

#### 4.2.4. Unified Contribution Score

Since $u_{i}$, $d_{i}$, and $q_{i}$ are measured on different scales, they are normalized across all candidate clients through min–max normalization:

(12) $${\bar{u}}_{i}=\frac{u_{i}-u_{\min}}{u_{\max}-u_{\min}+\epsilon },\;{\bar{d}}_{i}=\frac{d_{i}-d_{\min}}{d_{\max}-d_{\min}+\epsilon },\;{\bar{q}}_{i}=\frac{q_{i}-q_{\min}}{q_{\max}-q_{\min}+\epsilon },$$

where ϵ is a small constant for numerical stability.

The final contribution score of client $c_{i}$ is then defined as $I_{i}=\theta_{u}{\bar{u}}_{i}+\theta_{d}{\bar{d}}_{i}+\theta_{q}{\bar{q}}_{i}$, where $\theta_{u},\theta_{d},\theta_{q}\geq 0$ and $\theta_{u}+\theta_{d}+\theta_{q}=1$. A larger $I_{i}$ indicates that the client has a higher contribution in terms of model perturbation, data heterogeneity, and validation utility, and may therefore require stronger unlearning.

### 4.3. Initial Unlearning Strength Assignment

After computing the contribution score of the target client $c_{u}$, DynamicFU converts it into an initial unlearning strength. Let $\eta_{u}^{\left(0\right)}$ denote the initial strength assigned to $c_{u}$. It is defined as

(13) $$\eta_{u}^{\left(0\right)}=\mathrm{clip}\left(\eta_{\min}+\left(\eta_{\max}-\eta_{\min}\right)I_{u},\;\eta_{\min},\;\eta_{\max}\right),$$

where $\eta_{\min}$ and $\eta_{\max}$ denote the minimum and maximum strength bounds, respectively, and clip(·) truncates the value into the specified interval.

The initial strength is further mapped to an initial target number of retraining rounds:

(14) $$R_{u}^{\left(0\right)}=\max\;\left(1,\mathrm{round}(R_{\mathrm{base}}\eta_{u}^{\left(0\right)})\right),$$

where $R_{\mathrm{base}}$ is the full shard-level training budget. Therefore, a client with higher contribution receives a larger initial unlearning budget.

### 4.4. Dynamic Shard Unlearning Procedure

After an unlearning request is issued, DynamicFU removes $c_{u}$ from its shard and retrains only the affected shard $s_{u}$ on the retained clients $C_{s_{u}}^{\setminus u}=C_{s_{u}}\setminus \left\{c_{u}\right\}$.

Let ${\hat{w}}_{s_{u}}^{\left(r\right)}$ denote the current model of the affected shard after *r* adaptive retraining rounds. In this work, the affected shard is initialized from random weights and then retrained round by round on $C_{s_{u}}^{\setminus u}$. The initial ensemble ${\tilde{W}}^{\left(0\right)}$ is obtained by replacing $w_{s_{u}}^{*}$ with the randomly initialized affected-shard model ${\hat{w}}_{s_{u}}^{\left(0\right)}$. After each round, the resulting shard model replaces the original model in the ensemble:

(15) $${\tilde{W}}^{\left(r\right)}={\left\{{\tilde{w}}_{s}^{\left(r\right)}\right\}}_{s=1}^{S},\;{\tilde{w}}_{s}^{\left(r\right)}=\left\{\begin{array}{cc} {\hat{w}}_{s_{u}}^{\left(r\right)}, & s=s_{u},\\ w_{s}^{*}, & s\neq s_{u}. \end{array}\right.$$

To decide whether more rounds are needed, DynamicFU maintains an online controller driven by two quantities: an online convergence signal and the global utility drop.

#### 4.4.1. Online Convergence Signal

During the online unlearning process, DynamicFU monitors a convergence-oriented signal derived from the retained data of the affected shard and the current ensemble behavior. After removing the target client from the shard-level training set, the adaptive controller determines whether the retained-only retraining process has reached sufficient convergence while maintaining global utility.

Let $g\left(x;W\right)=\sum_{s=1}^{S}f\left(x;w_{s}\right)$ denote the ensemble logit function. Let $V_{s_{u}}^{\setminus u}$ denote the retained validation set of the affected shard. We first define the retained validation loss at round *r* as

(16) $$L_{\mathrm{ret}}^{\left(r\right)}=\frac{1}{|V_{s_{u}}^{\setminus u}|}\sum_{\left(x,y\right)\in V_{s_{u}}^{\setminus u}}\ell \;\left(g(x;{\tilde{W}}^{\left(r\right)}),y\right),$$

where ℓ(·,·) denotes the classification loss applied to logits and labels.

For r≥1, the relative retained-loss improvement is then defined as

(17) $$G\left(r\right)=\frac{\max\;\left(0,L_{\mathrm{ret}}^{\left(r-1\right)}-L_{\mathrm{ret}}^{\left(r\right)}\right)}{L_{\mathrm{ret}}^{\left(r-1\right)}+\epsilon }.$$

To further capture whether the ensemble behavior has stabilized, we compute the inter-round prediction variation:

(18) $$\Delta_{\mathrm{stab}}\left(r\right)=\left|F1_{\mathrm{macro}}\;\left({\tilde{W}}^{\left(r\right)}\right)-F1_{\mathrm{macro}}\;\left({\tilde{W}}^{\left(r-1\right)}\right)\right|.$$

The online control signal is finally defined as $\mathrm{Ctrl}\left(r\right)=\omega_{G}G\left(r\right)+\omega_{S}\Delta_{\mathrm{stab}}\left(r\right)$, where $\omega_{G},\omega_{S}\geq 0$ and $\omega_{G}+\omega_{S}=1$.

A larger Ctrl(r) indicates that the retained-only retraining process is still evolving and may require additional rounds, whereas a smaller value suggests that the affected shard has approached a stable post-removal state.

#### 4.4.2. Utility Drop

To avoid excessive utility degradation, DynamicFU also monitors the validation utility drop relative to the original ensemble:

(19) $$\mathrm{Drop}\left(r\right)=\max\;\left(0,\;F1_{\mathrm{macro}}\left(W^{*}\right)-F1_{\mathrm{macro}}\left({\tilde{W}}^{\left(r\right)}\right)\right).$$

A larger value of Drop(r) indicates that additional retraining is harming retained-task utility.

#### 4.4.3. Dynamic Strength Update and Stopping Rule

After each adaptive round r≥1, the unlearning strength is updated as

(20) $$\eta_{u}^{\left(r\right)}=\mathrm{clip}\left(\eta_{u}^{\left(r-1\right)}+\rho \;\mathrm{Ctrl}\left(r\right)-\mu \;\mathrm{Drop}\left(r\right),\;\eta_{\min},\;\eta_{\max}\right),$$

where ρ controls how strongly the online convergence signal encourages additional retraining, and μ controls how strongly utility degradation suppresses further retraining.

The updated target round budget becomes

(21) $$R_{u}^{\left(r\right)}=\max\;\left(1,\;\mathrm{round}(R_{\mathrm{base}}\eta_{u}^{\left(r\right)})\right).$$

The procedure continues until one of the following conditions is met:

1. The current round number reaches the updated target budget and the online control signal satisfies $\mathrm{Ctrl}\left(r\right)\leq \tau_{\mathrm{ctrl}}$;
2. The validation utility drop exceeds a preset tolerance, i.e., $\mathrm{Drop}\left(r\right)\geq \tau_{\mathrm{drop}}$;
3. The maximum adaptive round budget $R_{\max}$ is reached.

Through this design, DynamicFU does not apply a fixed number of retraining rounds to every unlearning request. Instead, it adaptively increases or suppresses the retraining intensity according to the estimated client contribution and the observed unlearning–utility trade-off during the retained-only retraining process.

### 4.5. Algorithm Summary

Algorithm 1 summarizes the overall procedure of DynamicFU.
**Algorithm 1** DynamicFU for Client-Level Unlearning in Sharded Federated Learning**Require:** Trained shard ensemble $W^{*}=\left\{w_{1}^{*},\ldots ,w_{S}^{*}\right\}$     shard partition $\left\{C_{1},\ldots ,C_{S}\right\}$, target client $c_{u}$     contribution weights $\theta_{u},\theta_{d},\theta_{q}$     data-contribution weights $\lambda_{\mathrm{JS}},\lambda_{\pi }$     controller weights $\omega_{G},\omega_{S}$ and parameters ρ,μ     strength bounds $\eta_{\min},\eta_{\max}$, base budget $R_{\mathrm{base}}$, maximum budget $R_{\max}$     stopping thresholds $\tau_{\mathrm{ctrl}},\tau_{\mathrm{drop}}$**Ensure:** Unlearned ensemble ${\tilde{W}}^{\setminus u}$  1:Identify the affected shard $s_{u}$ containing $c_{u}$  2:Remove $c_{u}$ from $C_{s_{u}}$ and obtain $C_{s_{u}}^{\setminus u}$  3:Compute parameter, data, and performance contributions for all candidate clients  4:Normalize the three components across candidate clients  5:Extract the unified contribution score $I_{u}$ of the target client $c_{u}$  6:Compute the initial unlearning strength $\eta_{u}^{\left(0\right)}$  7:Compute the initial target rounds $R_{u}^{\left(0\right)}$  8:Initialize the affected shard model ${\hat{w}}_{s_{u}}^{\left(0\right)}$ from random weights  9:Construct ${\tilde{W}}^{\left(0\right)}$ by replacing only shard $s_{u}$10:Set r←011:**while** *r* does not satisfy the stopping condition **do**12:      r←r+113:      Retrain the affected shard for one round on $C_{s_{u}}^{\setminus u}$14:      Construct ${\tilde{W}}^{\left(r\right)}$ by replacing only shard $s_{u}$15:      Compute Ctrl(r)16:      Compute Drop(r)17:      Update the unlearning strength $\eta_{u}^{\left(r\right)}$18:      Update the target round budget $R_{u}^{\left(r\right)}$19:**end while**20:Output ${\tilde{W}}^{\setminus u}\leftarrow {\tilde{W}}^{\left(r\right)}$

## 5. Experimental Setup

### 5.1. Datasets and Preprocessing

We use two representative IIoT intrusion-detection benchmarks: X-IIoT [32] and the network intrusion-detection subset of TON-IoT [33]. These datasets provide traffic records collected or constructed for IoT/IIoT security evaluation and therefore serve as practical proxies for industrial edge monitoring and networked cyber-physical environments.

Raw traffic records are transformed into numerical feature vectors before federated training. Identifier-type and highly instance-specific fields, including timestamp-, address-, and port-related attributes, are removed when necessary. Numerical features are normalized, while categorical features are encoded according to their cardinality. Each dataset is then split into training, validation, and test subsets, with the global validation and test sets fixed across all experiments.

To simulate heterogeneous industrial environments, the training split is partitioned into 50 federated clients using Dirichlet-based label-distribution partitioning. Three non-IID settings are considered, with α=0.1, α=0.5, and α=1.0, where smaller α denotes stronger statistical heterogeneity. The 50 clients are further grouped into 10 shards, and the same shard assignment is used throughout training, retraining, and unlearning.

### 5.2. Implementation Details

Unless otherwise specified, the same experimental settings are used across datasets to ensure a fair comparison. The number of clients is set to 50, the number of shards is set to 10, and each shard is trained for 20 communication rounds. In each round, all clients within the shard participate in training. Each selected client performs 2 local epochs using Adam with a learning rate of $1\times 10^{-2}$. The random seed is fixed to 42 for all experiments.

For the classifier architecture, we use multilayer perceptrons (MLP) whose input dimension is determined by the processed feature space of each dataset. For X-IIoT, a lightweight MLP is adopted, while for TON-IoT a slightly deeper MLP is used to match the higher feature complexity. The batch size is selected according to dataset scale and training stability, and all methods on the same dataset use the same architecture and optimization settings.

For DynamicFU, the contribution-score weights, data-contribution coefficients, and controller parameters are selected through preliminary validation and then fixed across all datasets, non-IID settings, and unlearning cases. No dataset-specific or client-specific retuning is performed.

For the online controller, the retained validation set of the affected shard is used to compute the retained-loss improvement term, while the global validation set is used to measure macro-F1 variation and utility drop. Therefore, the adaptive stopping behavior of DynamicFU depends only on retained data and current model behavior, without requiring access to a full-retrained oracle during online unlearning.

### 5.3. Construction of Unlearning Cases

For each dataset and each non-IID setting, we first train the original shard-based federated ensemble. Based on the trained ensemble, we then compute the contribution score of every client using the mechanism described in Section 4. The clients are ranked by their contribution scores and divided into three representative levels, namely high-contribution, medium-contribution, and low-contribution groups.

To avoid drawing conclusions from a single manually selected client, we sample three representative clients from each contribution level. Therefore, each non-IID setting contains 9 client-level unlearning cases, and each dataset contains 27 unlearning cases in total across the three heterogeneity settings.

For every unlearning case, the target client is removed from its shard and the following methods are compared:

1. FedAvg, the original shard ensemble trained with FedAvg and used only as a no-unlearning reference;
2. Full Retrain, which retrains all shard models from scratch after removing the target client and serves as the strongest retraining-based offline reference;
3. FedEraser, a replay-based federated unlearning baseline that reconstructs the unlearned model using stored historical update information;
4. Rapid Retraining, an accelerated retraining baseline that applies a reduced fixed retraining budget after client removal;
5. Affected-Shard Full Retrain, which retrains only the affected shard from scratch after removing the target client while keeping all unaffected shard models unchanged;
6. DynamicFU, the proposed adaptive shard unlearning method.

### 5.4. Evaluation Protocol

We evaluate all methods from three complementary perspectives: retained-task utility, unlearning effectiveness, and efficiency.

For retained-task utility, we report classification accuracy and F1-score on the global test set. Since the evaluated IIoT benchmarks are class-imbalanced, F1-score is reported together with accuracy to provide a more balanced assessment of predictive quality after unlearning. For the main comparison tables, accuracy and F1-score are reported as mean ± standard deviation over the evaluated unlearning cases.

For unlearning effectiveness, we evaluate how closely the unlearned model approaches the Full Retrain reference. Specifically, we compare the methods using performance gaps relative to Full Retrain, membership inference attack (MIA) AUC, and parameter-space discrepancy. In this work, the core criterion is proximity to Full Retrain: smaller predictive gaps, smaller privacy-related gaps, and smaller parameter-space distances indicate that the unlearned model more closely matches the retrained reference and therefore achieves more complete client removal. The gaps ΔAcc. and ΔF1 are computed as absolute differences from Full Retrain. For MIA AUC, values closer to 0.5 indicate weaker membership distinguishability.

For efficiency, we report unlearning runtime, speedup over Full Retrain, and the number of adaptive retraining rounds used by DynamicFU. The speedup is computed as $\mathrm{Speedup}=T_{\mathrm{full}}/T_{\mathrm{method}}$, where $T_{\mathrm{full}}$ and $T_{\mathrm{method}}$ denote the runtime of Full Retrain and the compared method, respectively.

For Affected-Shard Full Retrain and DynamicFU, the reported runtime counts only the shard-level retraining stage triggered after the unlearning request. For DynamicFU, this reflects the actual cost of adaptively retraining the affected shard under the online controller defined in Section 4. The Full Retrain model is constructed only as an offline reference for effectiveness evaluation and is not used by the online controller of DynamicFU. Therefore, it is not included in the reported runtime of the proposed method.

Unless otherwise specified, all compared methods under the same dataset and non-IID setting share the same train/validation/test split, the same client partition, the same shard assignment, and the same pretrained shard ensemble. Therefore, the comparison isolates the effect of the post-training client-removal strategy itself.

## 6. Experimental Results and Analysis

This section reports the experimental results of DynamicFU on X-IIoT and TON-IoT under three Dirichlet-based non-IID settings, namely, α=0.1, α=0.5, and α=1.0. Unless otherwise specified, all values are averaged over 9 client-level unlearning cases for each α, where the target clients are drawn from high-, medium-, and low-contribution groups. The analysis focuses on five aspects: overall retained-task utility and unlearning behavior, proximity to the Full Retrain reference, adaptive behavior across contribution groups, computational efficiency, and sensitivity to online-controller weights.

### 6.1. Overall Results

Table 1 and Table 2 summarize the main results on X-IIoT and TON-IoT, respectively. In response to the need for broader comparison with recent federated unlearning methods, we include FedEraser and Rapid Retraining in addition to FedAvg, Full Retrain, Affected-Shard Full Retrain, and DynamicFU. FedAvg is included only as a no-unlearning reference to show the behavior of the original model without any post-request removal. Since FedAvg does not execute an unlearning stage, its efficiency entry is reported as “–” rather than as a numerical round count.

In these tables, ΔAcc. and ΔF1 denote the absolute predictive gaps from Full Retrain. Smaller values indicate that the corresponding method is closer to the retraining-based reference in terms of retained-task utility. The $L_{2}$ column reports the parameter-space distance between each compared model and the Full Retrain model. Since Full Retrain itself is used as the reference model for this distance calculation, its own $L_{2}$ distance is not applicable and is therefore marked as “–”. For FedEraser, the entry “replay” in the rounds column indicates that no additional local retraining round is performed during unlearning; instead, the unlearned model is reconstructed by replaying stored historical update information. However, this replay-based advantage is achieved at the cost of maintaining historical updates, which introduces additional storage overhead that is not reflected by the round count alone.

In Table 1 and Table 2, retained-task utility is reported with mean and standard deviation to make the comparison more informative than single-run accuracy values. We avoid bolding the retained-utility columns because the purpose of federated unlearning is not simply to maximize post-removal accuracy but to approach the Full Retrain reference while reducing the cost of client removal. Boldface is therefore reserved for the most relevant proximity or efficiency indicators among approximate unlearning methods.

Several observations can be drawn from Table 1 and Table 2. First, the retained utility of most methods is numerically close, especially on X-IIoT. This explains why accuracy alone is not sufficient to demonstrate the advantage of DynamicFU. The benefit becomes clearer when accuracy and F1-score are interpreted together with the gaps from Full Retrain, MIA AUC, parameter-space distance, and required rounds. DynamicFU keeps the predictive gaps small while substantially reducing the post-request retraining budget.

Second, the additional baselines show different trade-offs. FedEraser avoids local retraining through stored-update replay and therefore does not require additional local optimization rounds during unlearning. However, this efficiency comes with an important storage-side cost because historical client or server update information must be retained for later replay. Such storage overhead may become non-negligible in long-running IIoT federations with many communication rounds, clients, or repeated unlearning requests. Rapid Retraining uses a fixed reduced budget and can preserve utility well, but its budget is not adapted to the contribution of the removed client. Affected-Shard Full Retrain provides a strong shard-level reference, yet it always uses the full shard retraining budget. In contrast, DynamicFU connects the estimated client contribution with the actual unlearning budget, which allows it to reduce unnecessary correction for lower-contribution removals while still allocating more effort to influential clients.

Third, the interpretation of the $L_{2}$ column should be tied to its reference role. Full Retrain is the target reference used to compute parameter-space proximity, so its own distance is undefined rather than zero-valued in the comparison table. The “–” entry avoids presenting the reference model as a competing approximate method in this column. Among the approximate unlearning methods, DynamicFU achieves the smallest $L_{2}$ distance in all X-IIoT settings and all TON-IoT settings. This indicates that the proposed adaptive correction not only preserves observable utility but also moves the model closer to the retraining-based reference in parameter space.

Fourth, on TON-IoT, the strongest heterogeneity setting α=0.1 is visibly more challenging. DynamicFU attains competitive retained accuracy and F1-score under this setting while still requiring fewer post-request rounds than the retraining-based alternatives. Under α=0.5 and α=1.0, all methods become very close in retained-task utility, suggesting that the shard-based approximation becomes easier when client heterogeneity is moderate or mild.

Finally, among methods that perform post-request optimization, DynamicFU generally requires the smallest or near-smallest number of rounds while maintaining retraining-like behavior. This directly reflects the core design of the proposed framework: the correction budget is assigned adaptively rather than fixed uniformly for all unlearning requests.

### 6.2. Case-Level Parameter Proximity

**Figure 2:**
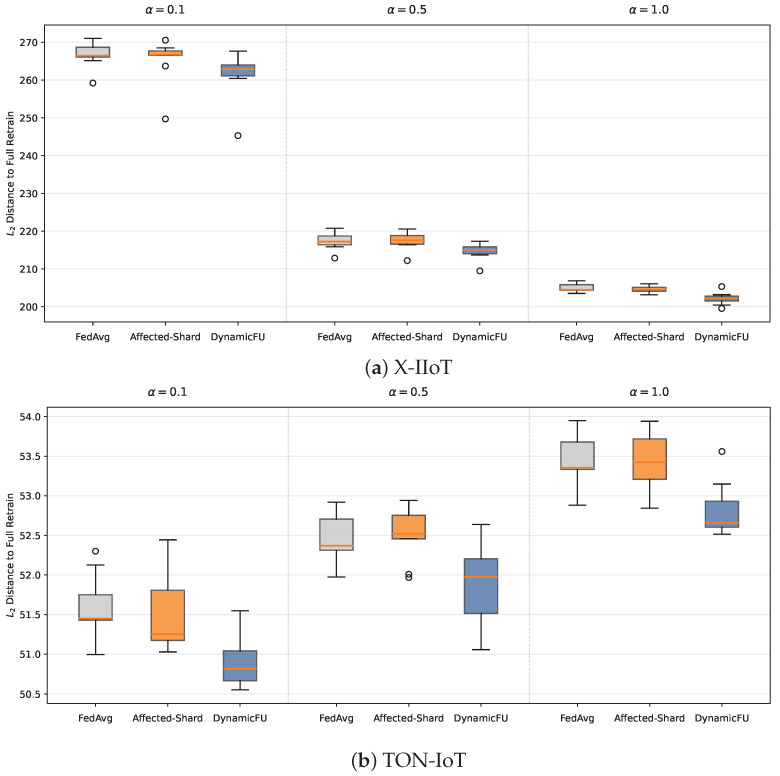
Pairwise $L_{2}$ distance to Full Retrain under different non-IID settings. A smaller distance indicates that the unlearned model is closer to the retrained reference in parameter space. Colors distinguish different compared methods, circles indicate outliers, boxes show the interquartile range, and horizontal lines inside the boxes indicate medians.

The main tables already report the average $L_{2}$ distance to Full Retrain for each method. To further examine whether this proximity is consistent across individual unlearning cases rather than only reflected by averaged values, Figure 2 presents the case-level distribution of parameter-space distances under different non-IID settings.

Compared with the averaged $L_{2}$ values in Table 1 and Table 2, the boxplots provide a more fine-grained view of the stability of each approximate unlearning strategy. On X-IIoT, the distances are larger in absolute magnitude because of the larger model and data scale, but DynamicFU remains consistently close to the retraining-based reference across the evaluated unlearning cases. This indicates that its parameter-level proximity is not caused by a few favorable target clients.

On TON-IoT, the distance distributions are more compact, suggesting that the retraining approximation is generally easier at the parameter level. Under the strongest heterogeneity setting, the spread becomes more visible, which is consistent with the greater difficulty of client removal when local distributions are highly skewed. Nevertheless, DynamicFU still maintains a stable distribution of distances to Full Retrain.

Overall, Figure 2 complements the main tables by showing the robustness of retraining proximity across individual unlearning trials. This case-level evidence supports the claim that DynamicFU achieves not only competitive average performance but also stable parameter-space approximation to the retraining-based reference under heterogeneous IIoT settings.

### 6.3. Adaptive Unlearning Across Contribution Groups

A central objective of DynamicFU is to avoid assigning the same unlearning budget to all clients. In heterogeneous IIoT environments, clients may differ substantially in data volume, class composition, data diversity, and model contribution. Removing a high-contribution client may therefore require a stronger post-removal correction than removing a marginal one. To verify whether the proposed adaptive mechanism behaves as intended, we group the target clients selected for unlearning into high-, medium-, and low-contribution groups according to their estimated contribution scores.

Table 3 summarizes the mean contribution score, assigned unlearning rounds, runtime, and speedup across the three contribution groups. The results show a clear monotonic trend on both datasets: high-contribution clients receive more unlearning rounds, medium-contribution clients receive an intermediate budget, and low-contribution clients receive the fewest rounds. This confirms that DynamicFU does not reduce computation indiscriminately. Instead, it allocates additional correction effort only when the target client is estimated to have a stronger contribution to the trained ensemble.

**Figure 3:**
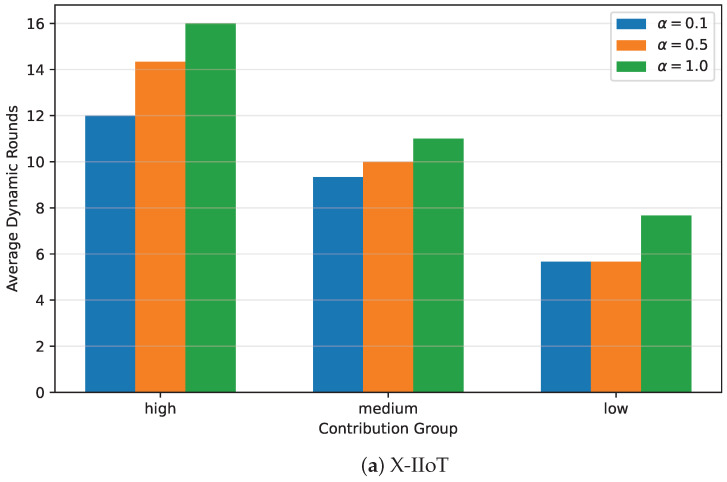

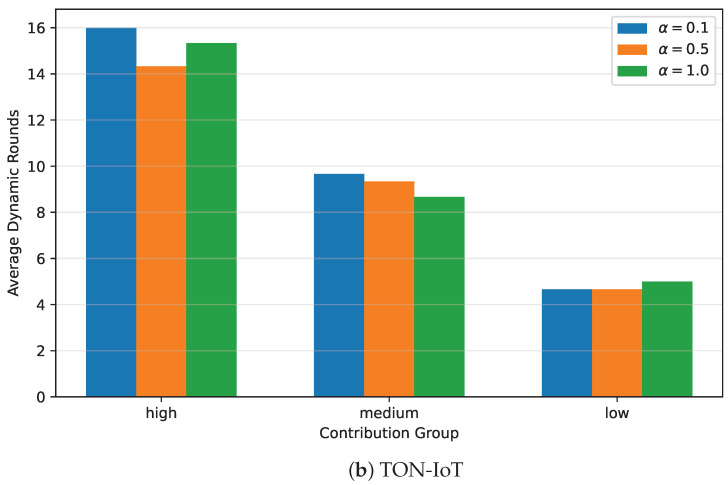
Adaptive unlearning rounds across high-, medium-, and low-contribution client groups.

Figure 3 provides a more detailed view of this adaptive behavior under different non-IID settings. On both X-IIoT and TON-IoT, the average number of dynamic unlearning rounds decreases from the high-contribution group to the low-contribution group. The trend is particularly pronounced on TON-IoT, where the high-contribution group consistently requires a much larger correction budget. This behavior is well aligned with the design motivation of DynamicFU: client-level unlearning in non-IID IIoT environments should be sensitive to uneven client importance.

### 6.4. Efficiency Analysis

Efficiency is one of the primary motivations for approximate federated unlearning. Although Full Retrain provides the cleanest retraining-based target, it requires retraining after each unlearning request and is, therefore, expensive in practical IIoT deployments. DynamicFU instead performs a targeted post-removal correction whose length is determined by the estimated contribution of the target client.

**Figure 4:**
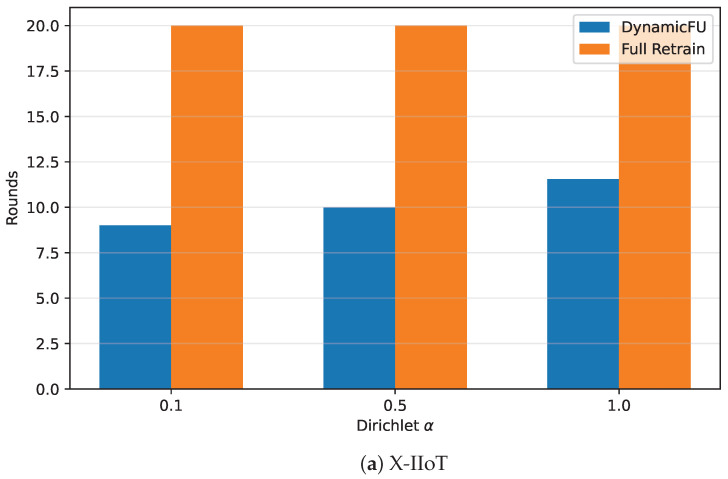

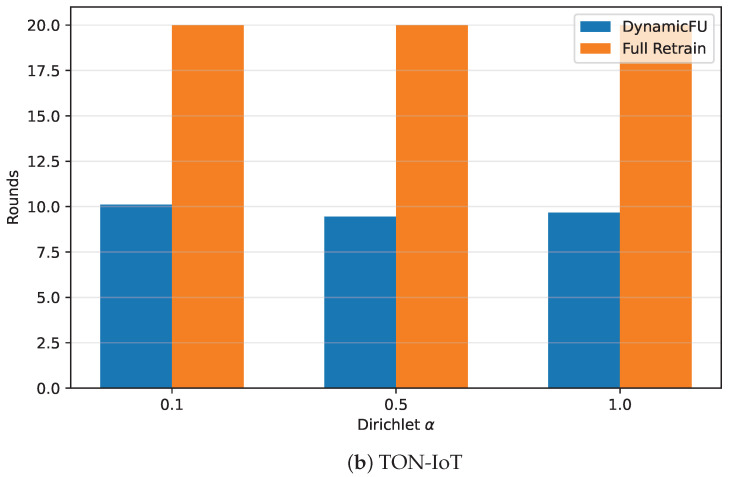
Average unlearning rounds of DynamicFU and Full Retrain under different non-IID settings.

Figure 4 compares the average number of rounds required by DynamicFU and Full Retrain. Since Full Retrain always uses the full 20-round retraining budget, its cost is fixed across datasets and non-IID settings. By contrast, DynamicFU requires only about half of that budget on average. On X-IIoT, the average dynamic rounds remain around 8 to 10 across the three non-IID settings. On TON-IoT, DynamicFU likewise requires fewer than 10 rounds on average. This confirms that the proposed method substantially reduces the post-request optimization cost while maintaining retraining-like behavior.

The contribution-group statistics in Table 3 further explain where this efficiency gain comes from. For high-contribution clients, DynamicFU deliberately allocates more rounds to avoid insufficient removal. For low-contribution clients, it sharply reduces unnecessary updates and therefore obtains much larger speedups. On X-IIoT, the speedup increases from 9.31× for high-contribution clients to 22.42× for low-contribution clients. On TON-IoT, the same trend is observed, with the speedup increasing from 6.88× to 22.89×. These results show that DynamicFU is not merely a shortened retraining procedure but an adaptive unlearning strategy that allocates computation where it is most needed.

### 6.5. Dynamic Online Control-Signal Trajectory

**Figure 5:**
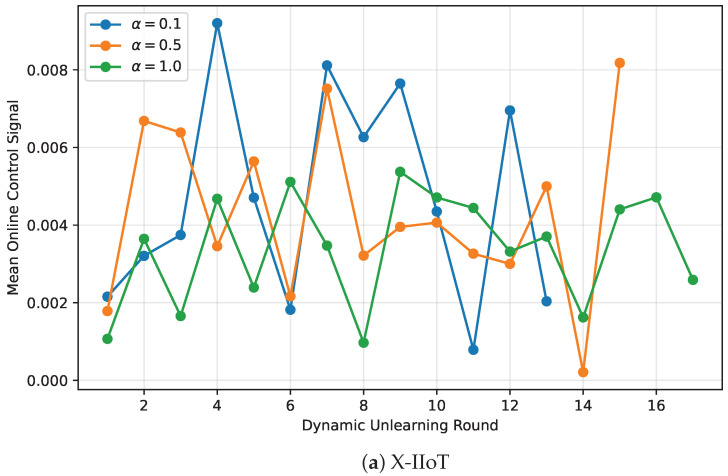

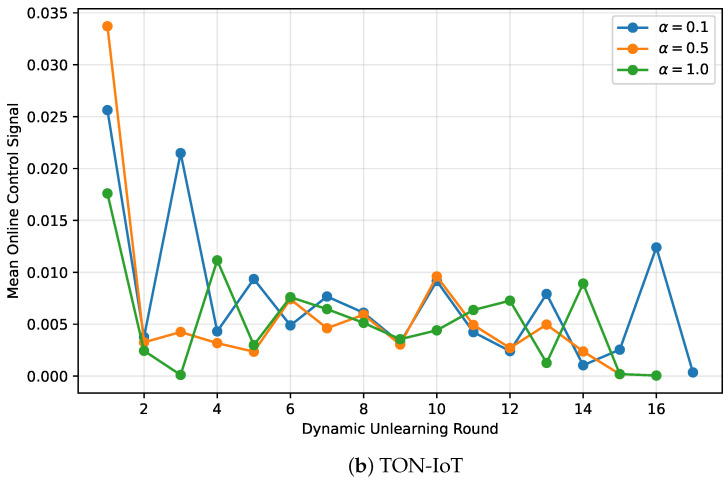
Online control-signal trajectory during dynamic unlearning. The curves show how the adaptive controller evolves as the assigned dynamic unlearning rounds proceed.

To further examine the internal behavior of DynamicFU, Figure 5 presents the mean online control-signal trajectory during dynamic unlearning. Unlike the final utility and proximity metrics, this figure provides a process-level view of how the adaptive controller evolves as additional shard-level correction rounds are performed.

On X-IIoT, the control-signal curves remain bounded and relatively smooth across all three non-IID settings. Although mild round-to-round fluctuations are still visible, the overall magnitudes stay low and stable, indicating that the online controller behaves in a controlled manner under different degrees of client heterogeneity. This observation is consistent with the main results, where DynamicFU preserves retained-task utility while requiring substantially fewer retraining rounds than Full Retrain.

On TON-IoT, the control-signal values are higher in magnitude and exhibit somewhat larger fluctuations, especially in the early rounds and under the strongest heterogeneity setting α=0.1. This behavior is reasonable because TON-IoT is a more challenging benchmark for adaptive post-removal optimization. Nevertheless, the trajectories remain bounded throughout the dynamic unlearning process and do not show unstable or divergent behavior. This suggests that the proposed online controller still provides a stable indication of whether further correction is necessary, even in more difficult heterogeneous settings.

Overall, the control-signal trajectories support the effectiveness of the proposed adaptive mechanism. Rather than assigning a fixed retraining budget to all client-removal requests, DynamicFU adjusts the correction process according to client contribution and the online evolution of the retained-only retraining objective. The resulting trajectories show that this adaptive process is stable, interpretable, and consistent with the efficiency gains reported in the final experimental results.

### 6.6. Sensitivity to Online-Controller Weights

The online controller in DynamicFU combines the retained-loss improvement term G(r) and the inter-round prediction-stability term $\Delta_{\mathrm{stab}}\left(r\right)$ through the weights $\left(\omega_{G},\omega_{S}\right)$. To examine whether the stopping behavior depends heavily on a specific weight choice, we conduct a compact sensitivity analysis by varying $\left(\omega_{G},\omega_{S}\right)$ over five settings: (0.2,0.8), (0.4,0.6), (0.5,0.5), (0.6,0.4), and (0.8,0.2).

Table 4 reports the observed ranges of stopping rounds, F1-score, and maximum utility drop across these weight settings. The results show that moderate changes in the online-controller weights lead only to limited variation in stopping rounds and retained F1-score. On X-IIoT, the F1 ranges remain within a narrow band for all three non-IID settings, and the maximum utility drop remains below 0.001. On TON-IoT, the stopping-round ranges are slightly wider under some settings, but the retained F1-score still varies only marginally. These results indicate that the controller is not overly sensitive to a single hand-picked weight pair. Rather, the stopping behavior is mainly governed by the joint trend of retained-loss convergence, prediction stability, and the utility-drop constraint.

## 7. Discussion

The experimental results indicate that client-level federated unlearning in IIoT should be evaluated through both retraining proximity and computational cost. DynamicFU remains close to retraining-based references in retained-task utility, privacy-related behavior, and parameter space while using substantially fewer post-request retraining rounds. The contribution-group analysis further shows that the adaptive budget is meaningful: high-contribution clients receive stronger correction, whereas low-contribution clients avoid unnecessary retraining. Compared with FedAvg, FedEraser, Rapid Retraining, and Affected-Shard Full Retrain, DynamicFU provides a more flexible balance between unlearning effectiveness and efficiency in heterogeneous IIoT environments.

These findings also clarify the role of the baselines. FedAvg is only a no-unlearning reference and should not be interpreted as a valid removal method, even when its retained-task utility is numerically close to that of unlearning methods. FedEraser provides an efficient replay-based strategy, but its approximation quality can vary under heterogeneous settings because the replayed historical updates are not explicitly adjusted according to the removed client’s current contribution. Rapid Retraining reduces the retraining budget, but the reduced budget is fixed rather than client-adaptive. Affected-Shard Full Retrain provides a strong shard-level retraining reference, but it still uses a fixed full budget for the affected shard. DynamicFU differs from these methods by explicitly estimating the removed client’s contribution and converting it into an adaptive post-removal correction budget.

The sensitivity analysis in Table 4 further suggests that the online stopping behavior is stable under moderate changes in the controller weights. Increasing $\omega_{G}$ gives more emphasis to retained-loss improvement, while increasing $\omega_{S}$ gives more emphasis to prediction-stability variation. The observed ranges show that neither term alone dominates the stopping decision under the tested settings. This supports the use of a fixed controller configuration after preliminary validation and indicates that the adaptive stopping rule is driven by the overall convergence and utility behavior rather than by a fragile weight choice.

Although the experiments use MLP classifiers, DynamicFU is not inherently tied to MLPs. The framework operates at the level of shard models, retained-client retraining, contribution estimation, and online stopping; therefore, the base learner can be replaced by recurrent models, such as LSTM or GRU, or by Transformer-based architectures when temporal packet sequences, flow histories, or event logs are available. In such cases, the parameter-contribution term can still be computed from model-parameter displacement while the data- and performance-contribution terms can be evaluated using the corresponding sequential validation behavior. However, more complex architectures may increase the cost of both contribution estimation and adaptive retraining. This makes the budget-aware nature of DynamicFU potentially more useful, because avoiding unnecessary retraining becomes even more important when each local update is expensive. At the same time, sequence models may introduce additional stability issues, such as longer convergence time, sensitivity to sequence length, and stronger dependence on temporal feature engineering. These aspects require dedicated empirical validation in future work.

From a deployment perspective, DynamicFU is particularly suitable for industrial federated systems in which unlearning requests may occur repeatedly, but full retraining is difficult to schedule. Since the proposed method retrains only the affected shard and determines the correction budget according to the estimated client contribution, it can reduce unnecessary computation for low-contribution removals while still allocating stronger correction to influential clients. This behavior is desirable in IIoT environments with limited maintenance windows, bandwidth constraints, and continuous service requirements. Moreover, the online controller relies on retained validation data and current model behavior rather than a full-retrained oracle, which makes the method more practical for post-deployment unlearning in industrial systems.

## 8. Conclusions

This paper investigated client-level federated unlearning in heterogeneous IIoT environments and proposed DynamicFU, a contribution-aware dynamic federated unlearning framework that estimates the target client’s contribution from parameter, data, and performance perspectives to adaptively determine the unlearning strength. Extensive experiments on X-IIoT and TON-IoT under multiple Dirichlet-based non-IID settings demonstrated that DynamicFU achieves a favorable balance between unlearning effectiveness and efficiency, remaining close to the Full Retrain reference in utility and privacy-related behavior while substantially reducing the computational cost of client removal. These results indicate that explicitly considering client contribution is an effective way to support practical, flexible, and cost-sensitive federated unlearning in heterogeneous industrial environments.

## Figures and Tables

**Table 1:** Main unlearning comparison on X-IIoT. Acc., F1, ΔAcc., ΔF1, and MIA AUC are reported in percentage form. Full Retrain is used as the retraining-based reference. The downward arrows indicate that smaller values are preferred.

α	Method	Acc. (%)	F1 (%)	ΔAcc. (%) ↓	ΔF1 (%) ↓	MIA AUC (%)	$L_{2}$ to Full ↓	Rounds ↓
0.1	FedAvg	97.437 ± 0.000	97.432 ± 0.000	0.006	0.002	50.47	266.73	–
	Full Retrain	97.443 ± 0.025	97.430 ± 0.025	0.000	0.000	50.43	–	20.00
	FedEraser	96.551 ± 2.397	96.531 ± 2.442	0.892	0.899	50.42	265.51	replay
	Rapid Retraining	97.449 ± 0.028	97.445 ± 0.037	0.006	0.015	50.41	269.27	10.00
	Affected-Shard	97.436 ± 0.021	97.429 ± 0.026	0.007	0.001	50.48	264.52	20.00
	**DynamicFU (ours)**	97.415 ± 0.024	97.413 ± 0.024	0.019	0.017	50.47	**260.95**	**8.33**
0.5	FedAvg	97.261 ± 0.000	97.246 ± 0.000	0.007	0.014	49.55	216.99	–
	Full Retrain	97.268 ± 0.039	97.260 ± 0.039	0.000	0.000	49.52	–	20.00
	FedEraser	97.173 ± 0.223	97.168 ± 0.224	0.095	0.092	49.97	216.89	replay
	Rapid Retraining	97.296 ± 0.015	97.291 ± 0.015	0.028	0.031	50.00	220.06	**10.00**
	Affected-Shard	97.272 ± 0.015	97.257 ± 0.016	0.004	0.003	49.55	217.60	20.00
	**DynamicFU (ours)**	97.234 ± 0.014	97.231 ± 0.022	0.034	0.029	49.55	**213.99**	10.11
1.0	FedAvg	97.141 ± 0.000	97.143 ± 0.000	0.017	0.010	50.21	203.36	–
	Full Retrain	97.158 ± 0.050	97.153 ± 0.051	0.000	0.000	50.22	–	20.00
	FedEraser	96.945 ± 0.317	96.938 ± 0.319	0.213	0.215	50.23	203.29	replay
	Rapid Retraining	97.237 ± 0.009	97.232 ± 0.009	0.079	0.079	50.27	206.14	10.00
	Affected-Shard	97.133 ± 0.015	97.128 ± 0.015	0.025	0.025	50.17	203.18	20.00
	**DynamicFU (ours)**	97.088 ± 0.015	97.093 ± 0.015	0.070	0.060	50.18	**201.25**	**9.11**

**Table 2:** Main unlearning comparison on TON-IoT. Acc., F1, ΔAcc., ΔF1, and MIA AUC are reported in percentage form. Full Retrain is used as the retraining-based reference. The downward arrows indicate that smaller values are preferred.

α	Method	Acc. (%)	F1 (%)	ΔAcc. (%) ↓	ΔF1 (%) ↓	MIA AUC (%)	$L_{2}$ to Full ↓	Rounds ↓
0.1	FedAvg	87.455 ± 0.000	85.009 ± 0.000	0.331	0.346	50.42	51.56	–
	Full Retrain	87.124 ± 0.512	84.663 ± 0.535	0.000	0.000	49.71	–	20.00
	FedEraser	86.177 ± 4.489	83.633 ± 4.283	0.947	1.030	49.05	51.56	replay
	Rapid Retraining	87.472 ± 0.121	85.026 ± 0.127	0.348	0.363	49.95	52.17	10.00
	Affected-Shard	87.410 ± 0.143	84.963 ± 0.150	0.286	0.300	50.45	51.52	20.00
	**DynamicFU (ours)**	87.521 ± 0.129	85.072 ± 0.159	0.397	0.409	50.67	**50.74**	**9.89**
0.5	FedAvg	84.772 ± 0.000	82.246 ± 0.000	0.027	0.019	49.75	52.58	–
	Full Retrain	84.799 ± 0.005	82.265 ± 0.005	0.000	0.000	49.68	–	20.00
	FedEraser	85.228 ± 1.636	82.722 ± 1.645	0.429	0.457	50.18	52.55	replay
	Rapid Retraining	84.768 ± 0.010	82.243 ± 0.009	0.031	0.022	50.10	53.26	10.00
	Affected-Shard	84.776 ± 0.012	82.261 ± 0.012	0.023	0.004	49.75	52.68	20.00
	**DynamicFU (ours)**	84.771 ± 0.010	82.236 ± 0.010	0.028	0.029	49.74	**52.38**	**9.44**
1.0	FedAvg	84.759 ± 0.000	82.244 ± 0.000	0.006	0.004	49.50	53.28	–
	Full Retrain	84.765 ± 0.021	82.240 ± 0.021	0.000	0.000	49.31	–	20.00
	FedEraser	85.503 ± 2.147	82.763 ± 1.546	0.738	0.523	50.65	53.23	replay
	Rapid Retraining	84.772 ± 0.009	82.247 ± 0.009	0.007	0.007	50.84	53.88	10.00
	Affected-Shard	84.770 ± 0.007	82.235 ± 0.007	0.005	0.005	49.49	53.24	20.00
	**DynamicFU (ours)**	84.765 ± 0.009	82.240 ± 0.009	0.000	0.000	49.49	**52.86**	**9.33**

**Table 3:** Adaptive behavior of DynamicFU across contribution groups.

Dataset	Contribution Group	Mean Contribution	Avg. Rounds	Runtime (s)	Speedup
X-IIoT	High	0.563	12.78	18.64	9.31
	Medium	0.360	9.00	10.92	16.35
	Low	0.166	5.78	7.84	22.42
TON-IoT	High	0.656	15.00	4.97	6.88
	Medium	0.322	9.11	2.88	12.90
	Low	0.079	4.56	1.62	22.89

**Table 4:** Compact sensitivity analysis of DynamicFU to online-controller weights. For each α, five settings of $\left(\omega_{G},\omega_{S}\right)$ are tested: (0.2,0.8), (0.4,0.6), (0.5,0.5), (0.6,0.4), and (0.8,0.2). The table reports the observed ranges across these settings.

Dataset	α	Rounds Range	F1 Range (%)	Max Utility Drop
X-IIoT	0.1	9.67–10.33	97.469–97.476	0.0002
X-IIoT	0.5	9.67–10.00	97.192–97.204	0.0004
X-IIoT	1.0	10.33–11.00	97.180–97.200	0.0006
TON-IoT	0.1	8.33–9.67	82.210–82.219	0.0000
TON-IoT	0.5	10.33–11.00	82.227–82.234	0.0003
TON-IoT	1.0	11.00–12.00	82.220–82.239	0.0001

## Data Availability

The datasets used in this study are publicly available. The X-IIoTID dataset can be accessed from its official repository at https://github.com/Alhawawreh/X-IIoTID (accessed on 2 June 2026) and its Kaggle page at https://www.kaggle.com/datasets/munaalhawawreh/xiiotid-iiot-intrusion-dataset (accessed on 2 June 2026). The TON-IoT dataset can be accessed from the official UNSW Research dataset page at https://research.unsw.edu.au/projects/toniot-datasets (accessed on 2 June 2026). The implementation code will be made available by the corresponding author upon reasonable request.
